# Extramedullary Soft Tissue Involvement and Discrepant Osseous Uptake on Tc-99m MDP and Ga-67 Citrate Scintigraphy in a Patient With Multiple Myeloma: A Case Report and Literature Review

**DOI:** 10.1097/MD.0000000000000995

**Published:** 2015-06-19

**Authors:** Szu-Ying Tsai, Shan-Ying Wang, Yu-Chien Shiau, Yen-Wen Wu

**Affiliations:** From Department of Nuclear Medicine, Far Eastern Memorial Hospital, New Taipei City, Taiwan (S-YT, S-YW, Y-CS, Y-WW); National Yang-Ming University School of Medicine, Taipei, Taiwan (Y-WW); and Department of Nuclear Medicine, National Taiwan University Hospital and National Taiwan University College of Medicine, Taipei, Taiwan (Y-WW).

## Abstract

Multiple myeloma (MM) is a plasma cell neoplasm with skeletal destruction which could also spread to extramedullary regions. Common diagnostic imaging modalities include skeletal radiography, computed tomography (CT), magnetic resonance imaging (MRI). Recently, PET/CT is proposed as an ideal tomographic tool for diagnosis and follow-up, but impending factors includes high cost, limited availability of cameras and radiotracers. Bone scan and gallium scan are usually considered of limited clinical value.

Herein, we present a 66-year-old Taiwanese man with MM, who was hospitalized to our hospital for bone pain control. Bone and gallium scintigraphies were obtained for bone pain and infection workup. However, unexpected features of discordant osseous uptake with high gallium-to-bone uptake ratio and extramedullary gallium uptake were noted which both indicated poor prognosis of MM. The patient then passed away due to rapid disease progression.

In conclusion, although gallium and bone scintigraphies are considered less sensitive for MM, combined use may be a good alternative for ^18^F-FDG PET/CT in evaluation of disease extent and prognosis, especially in high-risk patients or with suspicion of disease progression.

## INTRODUCTION

Multiple myeloma (MM) is a plasma cell neoplasm derived from bone marrow, which resulted in production of monoclonal immunoglobulin (M protein). Extensive skeletal destruction and bone pain are common presentations. Imaging modalities including skeletal radiography, computed tomography (CT), magnetic resonance imaging (MRI), and positron emission tomography (PET) play different roles in evaluation of bone lesions at diagnosis.^1^ Bone scintigraphy is usually not suggested since osteolytic lesions are with little bone reaction, and could usually cause false-negative results.^2^ Most MM lesions are restricted to bone marrow, but could also spread to extramedullary regions. Extramedullary involvement is a poor prognostic indicator,^3,4^ which could be detected by gallium scintigraphy.^5–7^ Recently, PET/CT with 2-deoxy-2-[fluorine-18]fluoro-d-glucose (^18^F-FDG) is proposed as an ideal tomographic tool for diagnosis and follow-up, but impending factors includes high cost, limited availability of PET cameras, production and delivery of positron emitting radiotracers. We present a case of relapsed MM with specific nuclear medicine imaging features, which might shed light to aid evaluation of disease status even without the assistance of PET/CT.

## CASE PRESENTATION

A 66-year-old Taiwanese man was diagnosed with stage IIIb IgA λ MM 2 years prior, in November 2012. His initial β2-microglobulin level was 5.64 mg/L (normal range 0.61–2.37 mg/L), lactate dehydrogenase (LDH) level was 194 U/L (normal range 135–225 U/L), serum calcium level was 15.2 mg/dL (normal range 8.5–10.5 mg/dL) and serum creatinine level was 2.05 mg/dL. Marked tumor reduction with very good partial response (a reduction of M-protein from 4280 to 368 mg/L, and not detected on electrophoresis)^8,9^ was achieved after 3 cycles of induction therapy with bortezomib, thalidomide, and dexamethasone (VTD).^9^ Therefore, primarily intended following autologous stem cell transplantation was postponed. Palliative zoledronic acid 4 mg per month and radiotherapy for bone lesions were arranged instead. He remained stable condition until October 2013 when abrupt elevation of paraprotein up to 1220 mg/L was noted. Maintenance therapy^9^ was commenced with thalidomide 100 mg daily, later shifted to single-agent therapy with lenalidomide 25 mg daily, but immediately tapered to 25 mg every other day due to unbearable skin irritation. The M protein then downgraded and his condition stabilized again.

**FIGURE 1:**
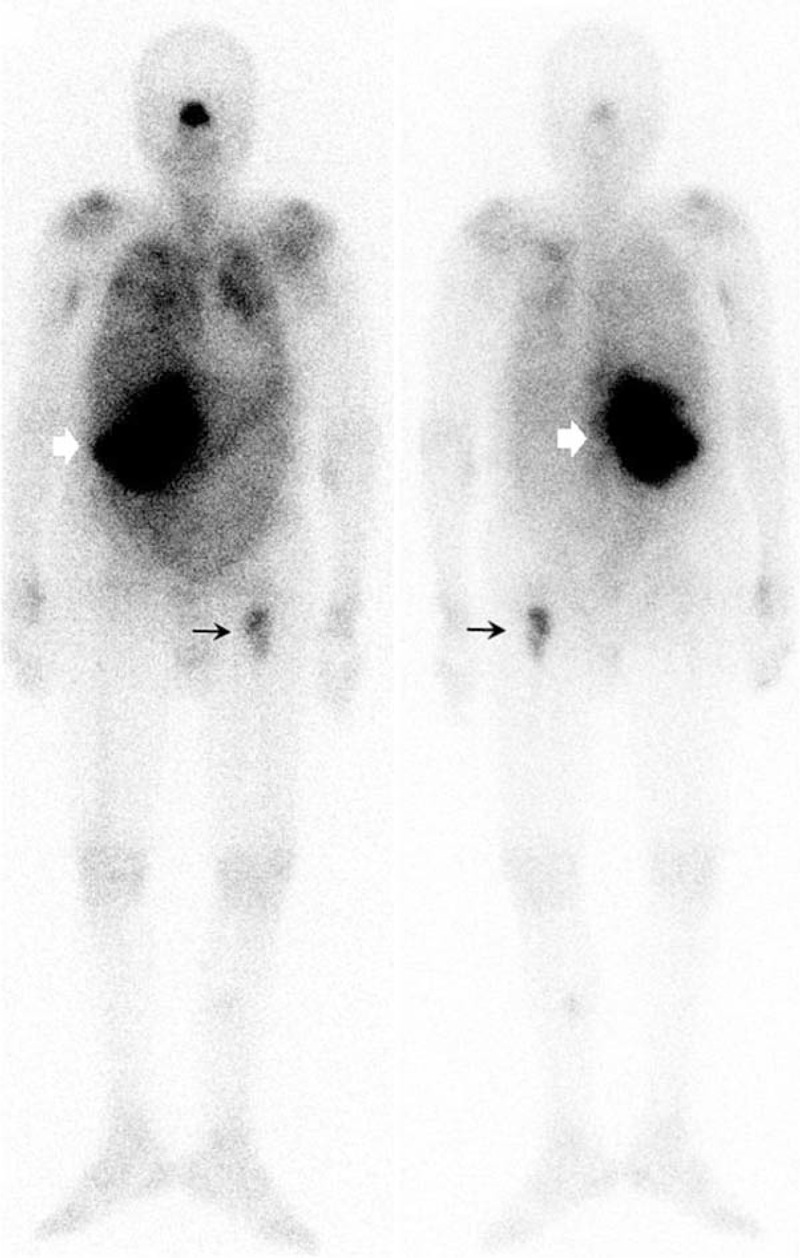
Ga-67 scintigraphy showed focal hot areas at left proximal femur (arrow), right abdomen (arrowhead) and paranasal region; also mild gallium avid uptake at bilateral lung fields, both shoulders, right proximal humerus, wrist and suspicious uptake at bilateral ribs and the sternum.

**FIGURE 2:**
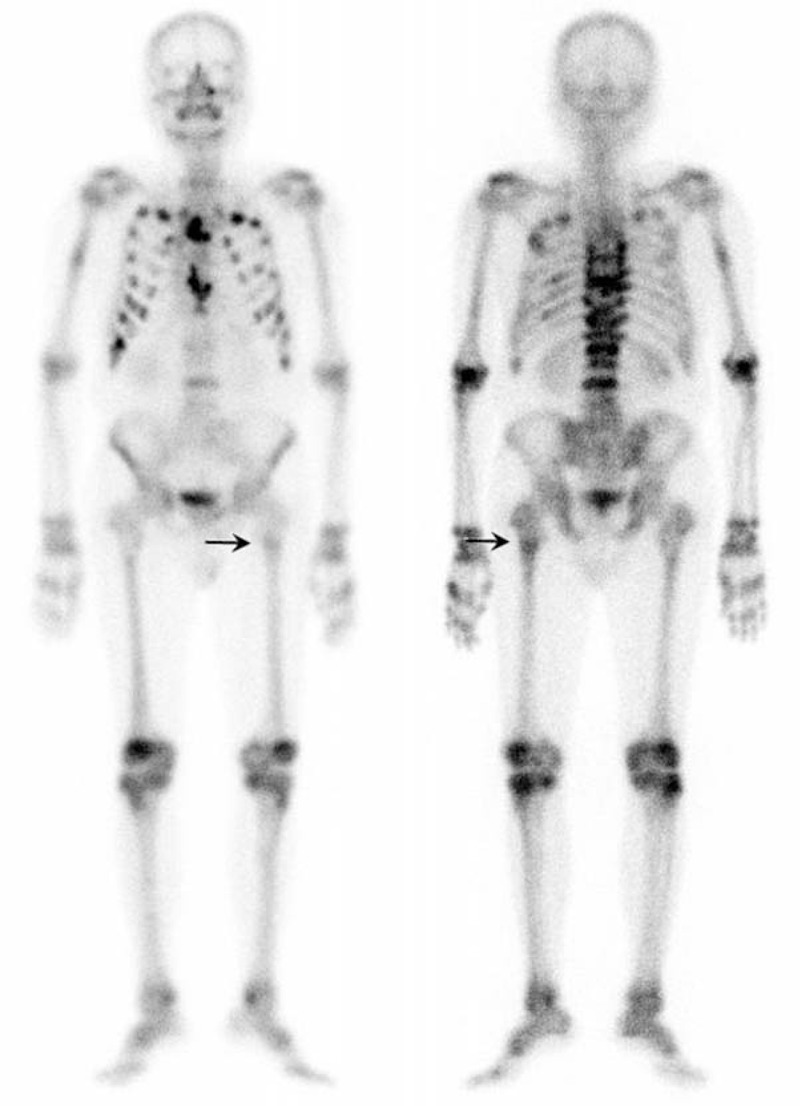
Whole-body Tc-99m MDP bone scan showed multiple intense hot areas at the sternum, thoracolumbar spines, bilateral ribs, and several mildly increased foci in the right proximal humerus and bilateral proximal femurs (arrow, left proximal femur).

**FIGURE 3:**
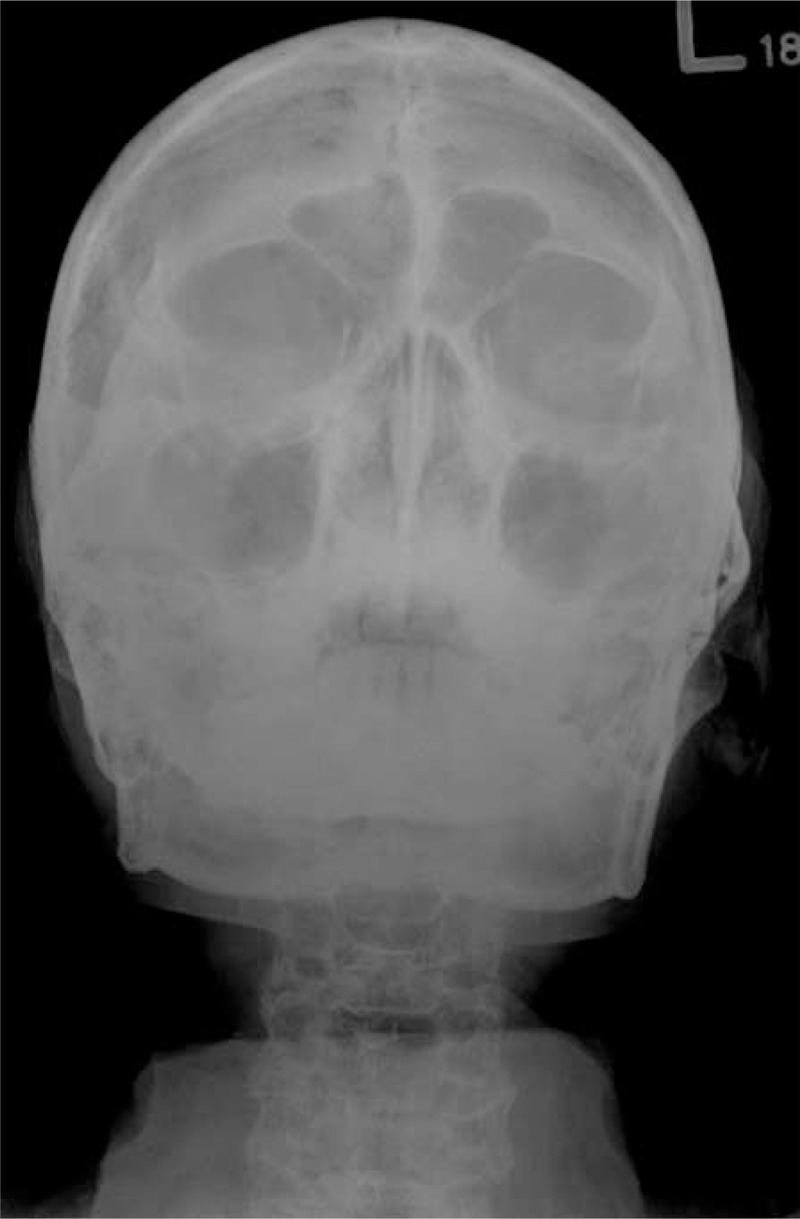
Water's view of skull X-ray was arranged for possible sinusitis, which showed clear paranasal sinuses, except multiple punch-out lesions on skull.

**FIGURE 4:**
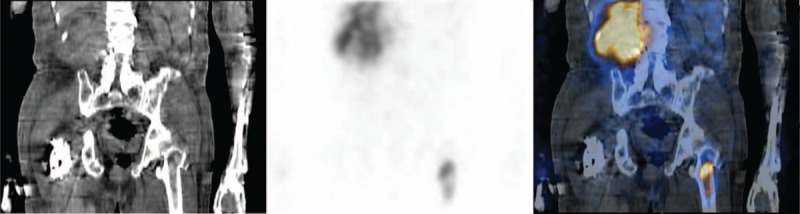
Regional SPECT/CT revealed soft tissue mass with intensely increased Ga-67 uptake at right abdomen and left proximal femur. Discordantly increased Ga-67 compared with Tc-99m MDP activities was noted in the left proximal femur.

**FIGURE 5:**
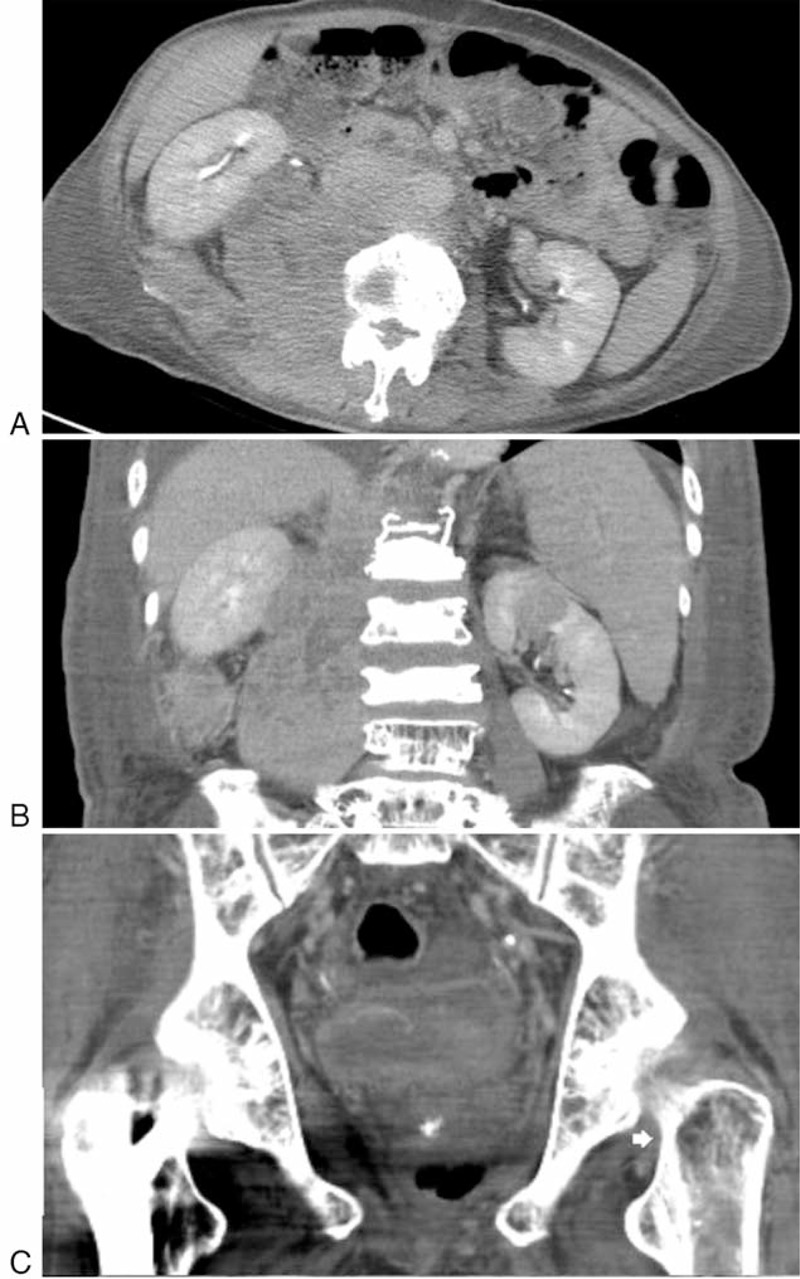
Abdominal CT following the Ga-67 scan. (A) Transverse and (B) coronal views showed a large right iliopsoas mass extending to the right peri-renal, posterior pararenal and prevertebral space, and (C) an obvious osteolytic lesion at left proximal femur (arrowhead). Biopsy from the right paraspinal region proved plasma cell myeloma, indicating extramedullary disease.

In July 2014, severe right hip pain developed with mild elevation of LDH level to 272 U/L. He then received palliative surgery with tumor excision and open reduction with internal fixation, followed by radiotherapy of right femur, which resulted in partial improvement. However, progressive unsustainable bilateral hip pain recurred in September 2014; therefore, he was hospitalized for pain control. During hospitalization, intermittent fever was noted. Physical examination revealed mildly coarse breath sounds bilaterally, and soft abdomen with normoactive bowel sounds. Serial cultures, including blood, urine specimens, and swap cultures from medical staffs’ hands, all disclosed methicillin-sensitive *Staphylococcus aureus* (MSSA). Leukocytosis gradually subsided (initially 12,520 cells/mm^3^, to 7290 cells/mm^3^) after culture-sensitive antibiotics, but low grade fever persisted. Transthoracic echocardiogram showed no definite vegetation. Therefore, gallium-67 scintigraphy was arranged to exclude right hip prosthesis infection. Intriguingly, subsequent gallium scan (Figure 1) showed no increased uptake in right hip. Unexpected evident discrepancy of osseous uptake between gallium scan and bone scan 3 months prior (just before the right hip surgery) in the left proximal femur (Figure 2). Intense gallium-avid hot areas in paranasal region and right abdomen were also noted. Water's view X film revealed multiple punch-out lesions at skull, but clear sinuses (Figure 3). Either myeloma involvement or abscess within abdomen was suspected (Figure 4). Abdominal CT disclosed a large right iliopsoas mass with extension to right peri-renal space, right posterior pararenal space, and prevertebral space (Figure 5), later biopsy proved plasmacytoma. Due to rapid deterioration, after 1 cycle of palliative chemotherapy with vincristine, doxorubicin, and dexamethasone (VAD), the patient decided hospice care and passed away 6 weeks later.

## DISCUSSION

For imaging of MM, conventional radiography is considered standard of reference, mainly used to identify lytic bone lesions.^1,10^ It is frequently underestimated disease severity due to little bone reaction in osteolytic lesions.^2^ In addition, it is of limited value in the detection of disease progression and treatment response. Newer technique PET/CT with ^18^F-FDG, which could provide tomographic information, is generally considered promising in both diagnosis and therapy assessment for follow-up,^11,12^ but is not routinely used, including Taiwan.

Traditional nuclear medicine modalities bone and gallium scintigraphies are usually considered insensitive for myeloma involvement. But in combination, high gallium uptake with normal or only slightly normal MDP uptake on bone scan suggest fulminant disease with poor prognosis.^13,14^ Ga-67 scan is more sensitive in detecting soft tissue lesions rather than bone involvements.^6^ It provides the advantage in evaluation of the extramedullary spread of myeloma cells,^5–7^ and detection of metabolically active lesions that often precede evidence of osseous destruction at conventional radiography.^1^

The mechanism of extramedullary involvement remains not well-established, with incidence reported 7% to 18% in newly diagnosed patients and 6% to 20% in relapsed patients, respectively,^15^ which is not uncommon. The existence of extramedullary spread is associated with poor prognosis even with aggressive therapy.^3,4^ When disease was in progression, it might also lead to prolonged fever.^16^

In conclusion, although Ga-67 and Tc-99m MDP scintigraphies are considered of limited value as routine use in MM. However, in settings as high-risk patients at diagnosis or suspicious disease progression, combined use of Ga-67 and Tc-99m MDP scintigraphy might be a good alternative for ^18^F-FDG PET/CT in the evaluation of disease extent and prognosis, and contribute to the development of an appropriate strategy.
